# Is a single lethal electric field threshold sufficient to characterize the lesion size in computational modeling of cardiac pulsed-field ablation?

**DOI:** 10.1016/j.hroo.2025.02.014

**Published:** 2025-02-22

**Authors:** Argyrios Petras, Gerard Amoros Figueras, Zoraida Moreno Weidmann, Tomás García-Sánchez, David Viladés Medel, Antoni Ivorra, Jose M. Guerra, Luca Gerardo-Giorda

**Affiliations:** 1 RICAM–Johann Radon Institute for Computational and Applied Mathematics, Linz, Austria; 2 Department of Cardiology, Hospital de la Santa Creu i Sant Pau, Universitat Autònoma de Barcelona, CIBER CV, Barcelona, Spain; 3 Department of Information and Communication Technologies, Universitat Pompeu Fabra, Barcelona, Spain; 4 Institute for Mathematical Methods in Medicine and Data-Based Modelling, Johannes Kepler University, Linz, Austria

**Keywords:** Cardiac ablation, Computer simulations, Pulsed field ablation, Electric field threshold, Mathematical modeling

## Abstract

**Background:**

Pulsed-field ablation (PFA) is a novel cardiac ablation technology based on irreversible electroporation (IRE). PFA computational models rely on identification of a lethal electric field threshold to predict the IRE area. However, the predicted lesion anisotropy ratios (width over depth) vary extensively among recent studies, and these discrepancies remain a subject of discussion.

**Objective:**

This work aims to evaluate the predicted lesion anisotropy ratios using a PFA computational model by applying it to an open-chest in vivo porcine model geometry.

**Methods:**

Six domestic swine underwent epicardial PFA applications using a previously described waveform protocol. Animals were killed at least 3 hours after the last ablation, and lesions were assessed using triphenyltetrazolium chloride (TTC) staining. Numeric simulations were performed on a segmented and meshed porcine thoracic computed tomography (CT) scan, mimicking the open-chest experimental setup.

**Results:**

The maximum width of all simulated lesions was observed at the epicardial surface. The anisotropy ratios (AR) of the experimental lesions were smaller than the simulated ones (AR experimental vs simulated, 1.0–1.7 vs 2–2.7; Q1–Q3 quartiles). Increasing the peak voltage resulted in larger lesions; however, the computational model clearly underestimated the increase in lesion depth compared with the experimental data.

**Conclusion:**

Our computational model shows that a single lethal electric field threshold is insufficient to accurately predict both lesion depth and width in cardiac PFA. Our study suggests that for the given PFA waveforms, a threshold between 270 and 500 V/cm provides satisfactory lesion depth estimations, and a higher threshold between 790 and 1000 V/cm better captures the lesion width.

## Graphical abstract

**Figure:**
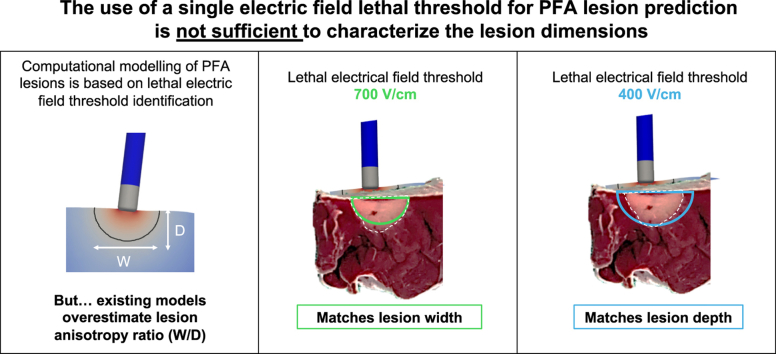

**Key findings**

▪ The use of a single lethal electric field threshold for the lesion identification is not sufficient to characterize the lesion shape (depth and width).
▪ In comparison with experimental results, matching the lesion width requires a higher threshold (790–1000 V/cm) with respect to matching the lesion depth (270–500 V/cm).
▪ Different PFA protocols feature different lethal thresholds for the identification of the lesion width or lesion depth.
▪ The existing models overestimate the lesion width-to-depth anisotropy ratio.

## Introduction

Catheter-based pulsed-field ablation (PFA) has emerged as a promising treatment for cardiac arrhythmias. This procedure involves the delivery of trains of monophasic or biphasic electrical pulses through catheter electrodes, either in a monopolar or bipolar configuration. The electrical pulses induce disruptions in the cellular membrane through electroporation, with the aim of introducing permanent damage via irreversible electroporation, while minimizing thermal effects,^1^ which is estimated to account for less than 2% of the total tissue damage.^2^ To ensure effective and safe outcomes, PFA protocols must be carefully designed to produce durable lesions while avoiding complications.^1^ However, optimizing these protocols is complex because of the number of parameters involved, with each waveform generating different lesions within the cardiac tissue.^2^^,^^3^

Modeling the multiscale mechanisms involved in PFA is challenging. Typical approaches in the field use an electrical potential equation to characterize the electric field produced by the applied voltage. The aim of such simple models is the identification of a lethal electric field threshold that can predict the extent of the irreversible electroporation (IRE) area, providing a simple and efficient approach for modeling such a complicated procedure. Previous studies have employed such computational models for the prediction of lesion sizes resulting from various PFA waveforms, exploring the impact of the pulse frequency,^4^ the electrode-to-tissue proximity,^5^ and the different types of bipolar protocols using multielectrode catheters,^6^ among others. In particular, for electrode-to-tissue proximity, newer experimental studies^7^^,^^8^ show contradictory results,^6^ although no computer simulations were used in these cases. Validation of these models typically involves comparing simulated lesion dimensions to those observed in experimental studies, using a single lethal electric field threshold. Different lethal thresholds have been reported in the literature for cardiac PFA, ranging from 268 V/cm to 2000 V/cm,^2^, ^3^, ^4^^,^^9^^,^^10^ using different specimens, catheters, and PFA protocols.

The current study aims to evaluate the predictive capability of a computational model, similar to that one proposed in Meckes et al,^4^ by applying it to data from an open-chest in vivo porcine experimental model.^11^ A detailed virtual porcine geometry is generated from a segmented thoracic computed tomography (CT) scan and adapted to replicate the experimental setup. A range of lethal electric field thresholds is explored to determine the IRE area that best correlates with the experimentally observed lesion dimensions.^11^ This study is the first to employ a full 3-dimensional porcine model geometry to assess the use of a lethal threshold for the lesion identification in PFA models.

## Methods

### Experimental setup

**Figure 1:**
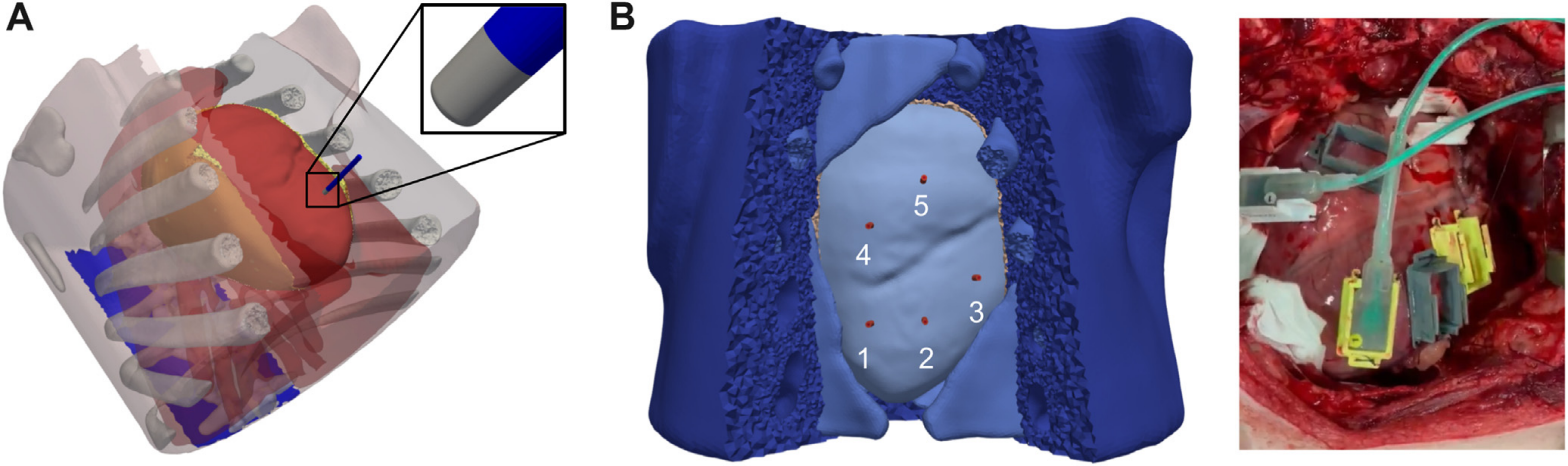
**A:** The computational model derived from the segmentation of a 57-kg swine CT scan. The model geometry was modified to mimic the open-thorax experimental setup. The catheter is placed on the epicardium of the ventricle, and the neutral electrode is positioned at the back. **B:** The location of the 5 electrode positions in the model geometry is based on the locations during the experimental setup.

This study involved 6 domestic swine (Landrace-Large White cross) weighing 57 ± 8 kg. Animals were anesthetized as detailed in Verma et al.^11^ The research reported in this paper adhered to ARRIVE and Guide for the Care and Use of Laboratory Animals guidelines. A median sternotomy was performed, and the pericardium was opened to expose the heart in a pericardial cradle. Two 3D-printed positioning frames were sutured at the epicardium and used as supports for the PFA applications (Figure 1B). Once the ablations were finished, 2 new locations sufficiently separated from the previous ablation points were selected, and the procedure was repeated again. At least 3 hours after the last ablation point was performed, animals were killed by inducing ventricular fibrillation, and cardiac tissue was immediately harvested for analysis. To assess the extent of acute lesion formation, we used triphenyl tetrazolium chloride (TTC) staining.

### PFA protocol

A custom-built generator (EPULSUS®-FBML1–5, Energy Pulse Systems, Lisbon, Portugal) capable of producing microsecond range biphasic pulses was used to deliver the PFA treatments. The waveform used is detailed in Meckes et al.^4^ Two different PFA groups were studied, corresponding to 2 different applied voltage levels: 1000 V and 2000 V (values are peak voltages). PFA was delivered unsynchronized from the electrocardiogram in a monopolar way between a focal electrode positioned in contact with the epicardium through the positioning frame previously described and a return patch attached to the back of the animal. Conductive electrocardiography gel was used to ensure good electrical contact. The focal electrode consisted of an 8F 3.5-mm tip ablation catheter (Thermocool® Smartouch®, Biosense Webster, Irvine, CA).

### Computational model

A thoracic CT scan (Aquilion One Vision CT Scanner; Canon Medical Systems, Otawara, Japan) of a 57-kg swine not submitted to the ablation procedure was segmented using Slicer3D software.^12^ The segmentation consists of the bones, lungs, blood vessels, 4-chamber heart, and pericardium. To mimic the open-chest experimental setup, the costal cartilage, sternum, and portions of the pericardium and torso were removed, as shown in Figure 1A and detailed in Supplementary Material 1. An 8F catheter with a 3.5-mm cylindrical tip electrode is placed at different positions on the epicardium of both the left and right ventricles (3 on the LV and 2 on the RV). Figure 1B shows the selected catheter positions in the simulation setup, corresponding to their locations during the experiments.

A 15 × 9 cm^2^ dispersive patch is positioned along the spine at the back of the geometry. A tetrahedral finite element mesh is created using Studio software from NumeriCor GmbH (Graz, Austria). The electric field is computed using a standard nonlinear potential equation, as described in the Supplementary Material of Meckes et al.^4^ Electrical conductivity parameters of the cardiac muscle are adjusted in electroporated areas, as detailed in Supplementary Material 1. Different field thresholds in the range 200–1000 V/cm are explored to model irreversible electroporation. The numerical simulations, implemented using the finite element method in FEniCSx.^13^

### Lesion assessment

**Figure 2:**
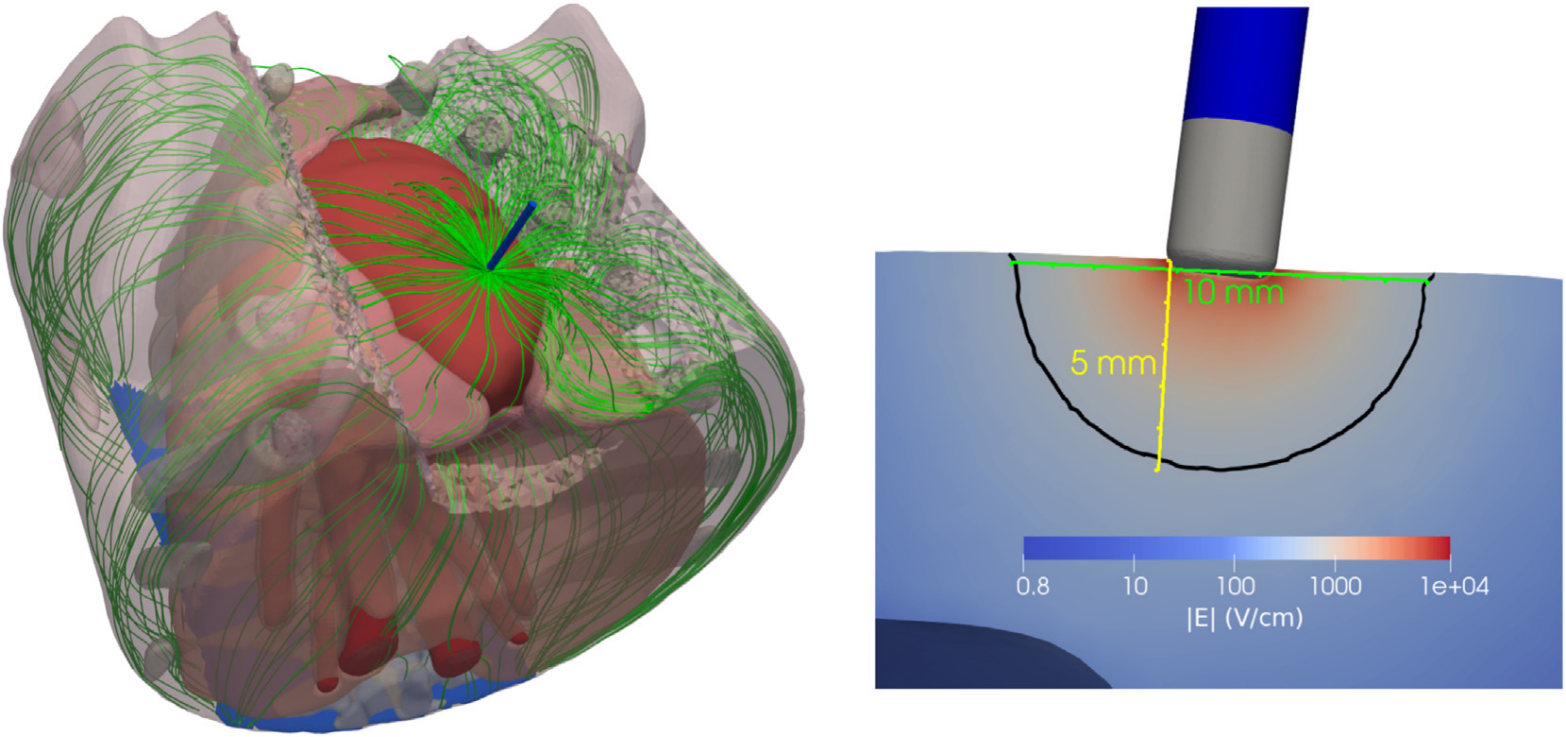
Left: The simulated electric field lines within the porcine torso, following the path from the active electrode to the dispersive patch. Right: The lesion depth (D, in yellow) and width (W, in green) as measured from a cross-section of the left ventricular wall.

Different electric field lethal thresholds are considered to identify the IRE area and assess the lesion extent. The lesion depth (D) and width (W) are measured on a cross-sectional plane at the midpoint of the catheter, following an approach similar to the standard histological assessment used in experimental studies. To assess lesion morphology of nontransmural lesions, we used the anisotropy ratio (AR), defined as the ratio of lesion width to depth. Although AR is a good indicator for the lesion morphology for nontransmural lesions, it becomes biased by the wall thickness when transmurality is achieved. To assess lesion morphology of transmural lesions, we used the lesion width. Figure 2 shows an example of the electric field distribution during PFA and the corresponding lesion measurements for a specific lethal threshold at the second considered position on the LV.

## Results

### Lesion morphology

The maximum width of all simulated lesions occurred at the epicardial surface, regardless of the selected lethal threshold, aligning with most of the experimental lesions. The experimental lesion morphology (TTC-stained section of lesions) and AR for all experimental lesions are shown in Supplementary Material 2. All experimental lesions on the LV were nontransmural, whereas some experimental lesions on the RV achieved transmurality. Regarding simulated lesions, all LV lesions were nontransmural, and all RV lesions achieved transmurality.

**Figure 3:**
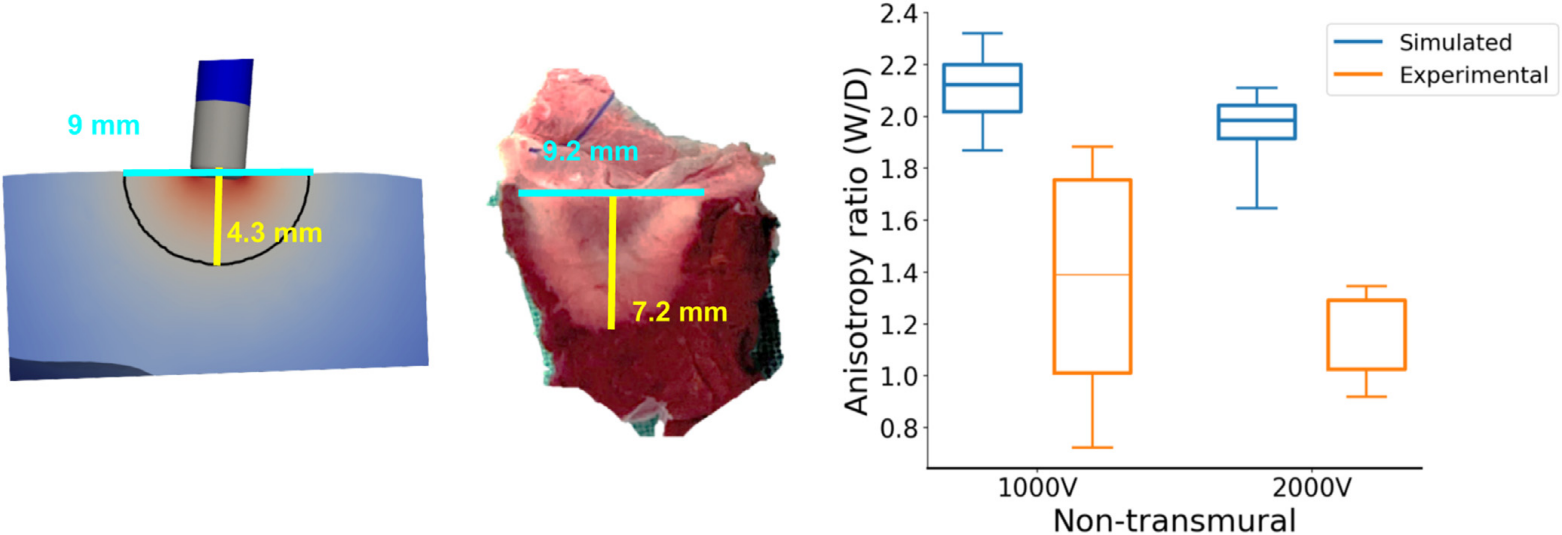
Sample nontransmural simulated and experimental lesions (left), and the corresponding anisotropy ratio (lesion width over lesion depth) of all the simulated and the experimental lesions for different peak voltages (right).

**Figure 4:**
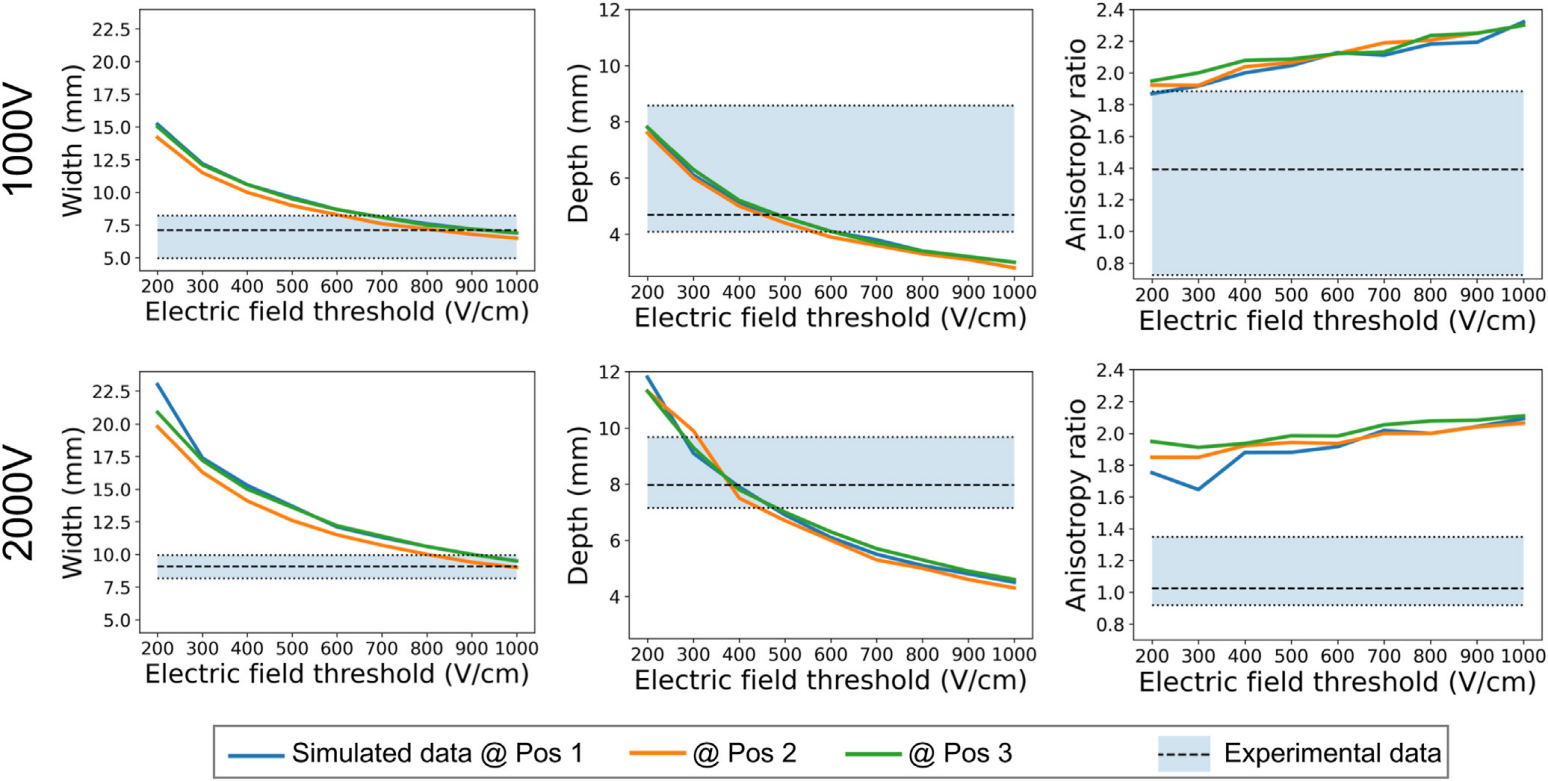
The predicted lesion depth, width, and anisotropy ratio for the 3 different electrode locations considered on the LV (non-transmural simulated lesions) for the 2 different peak voltages, by varying the electric field threshold. The shaded area presents the range of the nontransmural experimental lesion dimensions, and the dashed line is the median value of the experiments.

For nontransmural experimental lesions, the AR ranged from 0.7 to 1.9, irrespective of peak voltage, with a total median of 1.2, as shown in Figure 3. On the contrary, nontransmural simulated lesions featured an AR of at least 1.6. The smallest AR values are associated with lower lethal threshold choices and increase with higher thresholds (Figure 4). For typical thresholds between 300 and 600 V/cm, AR values range from 1.9 to 2.2.

For transmural experimental lesions, the lesion width ranged from 7.1 to 11.6 mm, whereas for the simulated lesions it ranged from 6.9 to 18.3 mm (Figure 4). Note the use of lesion width instead of the AR to quantify the changes in lesion morphology for transmural lesions. In Supplementary Material 1, we show the bias in AR to quantify the lesion morphology, because the AR of the simulated lesions is much larger than that of the experimental ones (1.9–6.1 vs 1.4–1.7). This difference stems from anatomic differences in the thickness of the RV of the model geometry derived from the segmentation of a swine, which was different from that used in the experiments (3–3.7 mm vs 5.2–7.2 mm).

### Impact of peak voltage

Increasing the peak voltage consistently resulted in larger lesion width and depth. For nontransmural lesions and the given PFA waveform, a 27% increase in median width and a 70% increase in median depth was observed for experimental lesions. The corresponding simulated lesions exhibited an increase in width between 38% and 51% and an increase in depth between 45% and 65%. These increases were independent of the chosen lethal threshold, showing a clear underestimation of the increase in lesion depth and an overestimation in the increase in the width.

In terms of transmural lesions, increasing the peak voltage from 1000 V to 2000 V in experimental lesions showed a 54% increase in median width. Simulated lesions exhibited an underestimated increase of 32% to 37%.

### Assessment of lesion dimensions

All electrode placements produced similar simulated lesion depths, regardless of the selected lethal threshold. Conversely, slightly smaller width values were consistently observed at position 2, as shown in Figure 4.

When compared with experimental data, thresholds between 200 and 600 V/cm produced lesion depths within the experimentally observed range for nontransmural simulated lesions at a peak voltage of 1000 V. The corresponding threshold range for 2000 V is 270 to 500 V/cm. In terms of the lesion width, thresholds in the range 580 to 1000 V/cm and 790 to 1000 V/cm provide values within the experimental range for 1000 V and 2000 V, respectively.

**Figure 5:**
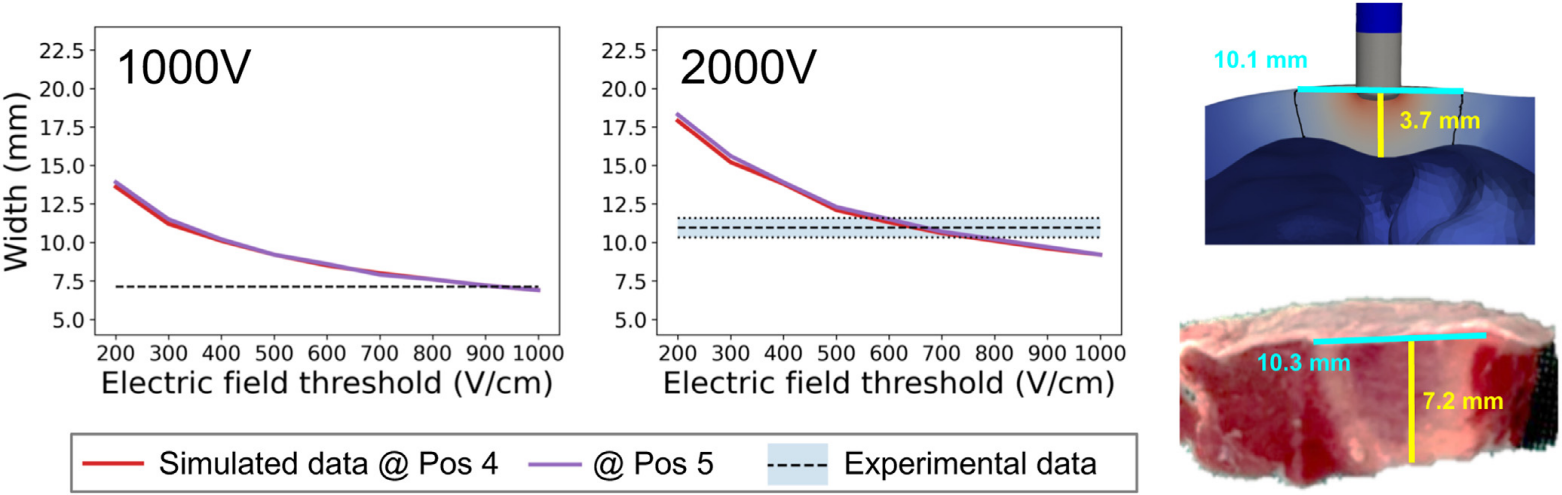
Sample transmural simulated and experimental lesions. Width for the 2 different electrode locations considered on the RV (transmural simulated lesions) for the 2 different peak voltages, by varying the electric field threshold. The shaded area presents the range of the transmural experimental lesion dimensions, and the dashed line is the median value of the experiments.

Transmural lesions provided a lesion width comparable to experimental data for a threshold of 900 V/cm and in the range of 550 to 760 V/cm for 1000 V and 2000 V, respectively, as shown in Figure 5.

## Discussion

This study is the first to use a full 3-dimensional porcine geometry, reconstructed from the segmentation of a thoracic CT scan of a 57-kg swine, to assess the use of a lethal threshold for lesion identification in PFA models.

### Lesion anisotropy ratio

Table 1 summarizes the anisotropy ratios from other experimental studies in the literature, including the electrode types, substrates, and ablation protocols used. The experimental data in Amorós-Figueras et al^11^ show a width-to-depth anisotropy ratio of between 0.7 and 1.9, which is comparable to that of other works that use a focal catheter and a neutral patch.^7^^,^^16^ Unlike atrial tissue, in which lesions often achieve transmurality because of the typically small thickness of the wall, and thus matching only the lesion width is sufficient for lesion characterization, in ventricular tissues it is essential to match both the lesion width and depth to properly identify the lesion morphology. The lesion morphology can be affected by the catheter geometry as shown in Table 1, where multielectrode catheters typically show a larger width-to-depth anisotropy ratio.

**Table 1:** The width-to-depth anisotropy ratio of the assessed lesions after the application of PFA in other experimental works available in the literature

Tissue type	AR	Animal model	Ablation type	Peak voltage (V)	Protocol type	References
Ventricles	3.5	Swine (∼56 kg), in vivo	Multielectrode basket	2200	Biphasic	^14^
	3.4–4.7	Swine (∼64 kg), in vivo	Focal lattice catheter	1300–2000	Biphasic	^15^
	1.9	Swine (∼74 kg), modified Langendorff	Focal multielectrode	1500	Biphasic	^5^
	0.9–1.1	Swine (55–75 kg), in vivo	Focal (single electrode + neutral patch)	Current 19 A, 22 A and 25 A	Biphasic	^16^
	0.6–1.7	Swine (57–74 kg), in vivo	Focal (single electrode + neutral patch)	Current 28 A and 35 A	Biphasic	^7^
	0.7–1.9	Swine (∼57 kg), open-chest	Focal (single electrode + neutral patch)	1000 and 2000	Biphasic 90 kHz	^11^

Our results demonstrate, for the first time, that computer models that use a single electric field lethal threshold cannot accurately reproduce both the experimentally observed lesion width and depth for nontransmural lesions. Other computer simulation works also show consistently higher anisotropy ratios of 2.42^17^ and 2.85^18^ by using a focal catheter.

### Lesion assessment using lethal thresholds

The standard computational modeling approach for identifying the IRE area and lesion size after PFA relies on the identification of an electric field lethal threshold.^3^ Numerous electric field lethal thresholds have been reported in the literature for estimating the IRE area after PFA, ranging from 268 V/cm to 2000 V/cm, each corresponding to models validated against experimental data.^2^, ^3^, ^4^^,^^9^^,^^10^ These thresholds may be valid for different experimental setups, ablation protocols, and delivery modalities. Our results demonstrate that a single threshold, used for the open-chest experimental setup with a focal catheter and neutral electrode, fails to accurately capture both lesion depth and width. This result aligns with the work of existing literature on skeletal anisotropic muscle, which suggested that different electric field lethal thresholds are needed to identify the surface lesion aspect ratio,^19^^,^^20^ showing that the angulation of the electric field has a direct impact on the lesion shape. In particular, we show that even for a fixed angle of the electric field, a single lethal threshold fails to capture the lesion width-to-depth anisotropy ratio for nontransmural lesions on ventricular tissues.

Additionally, we suggest that, unlike what has been reported in Meckes et al,^9^ different thresholds are required to predict the lesion depth at different peak voltages. Therefore, the results from such models cannot be generalized to predict lesion morphology across other PFA protocols (see also Table 1).

### Impact of the relative heart–electrode size

The experimental setup in Amorós-Figueras et al^11^ is comparable to that in García-Sánchez et al,^4^ both involving an open-chest configuration with PFA delivered through a focal catheter and a neutral electrode, though García-Sánchez et al^4^ used Sprague-Dawley rats and Amorós-Figueras et al^11^ used domestic swine. The same pulse frequency of 90 kHz was applied, with the peak voltage adjusted according to the size of the animals. Despite these similarities, the lesions produced in García-Sánchez et al^4^ were significantly more anisotropic compared to those in Amorós-Figueras et al.^11^ In García-Sánchez et al,^4^ the model successfully predicted the lesion size using a single lethal threshold. However, the lesions in Amorós-Figueras et al^11^ were more isotropic, likely because of the relative size of the heart and animal with respect to the ablating electrode. Even though we employed a detailed geometry based on a swine’s CT scan, the model using a single electric field threshold fails to accurately predict the shape of the more isotropic lesions in Amorós-Figueras et al.^11^

### Limitations

This study presents an anatomically detailed computational model geometry for PFA, though some simplification assumptions were made. Following the available models in the literature, the cardiac tissue's electrical conductivity is modeled as isotropic, despite the known influence of cardiac fibers, which result in higher conductivity along the fiber direction than across it. This anisotropy could affect lesion morphology and increase the anisotropy ratio predicted by the lethal threshold, an aspect worthy of investigation. In this direction, a recent study^2^ is the first to consider a computational model that includes anisotropic electrical conductivity parameters following the cardiac fibers on an idealized biventricular geometry: using lethal electric field thresholds that depend on the number of train of pulses in the waveforms, the study shows good agreement between the volumes of the simulated and the experimental lesions. Part of our ongoing work is to investigate the impact on lesion shape of fiber-driven anisotropic conductivity on our detailed 3-dimensional porcine model geometry.

The model geometry that we used for the simulations had a thinner RV wall than the mean depth of the RV lesions in the experimental pigs. Because of this, all the simulated lesion sizes in the RV were transmural, and therefore no change in depth could be assessed for the given PFA protocols. However, if we extrapolate the electrical field shape of the LV into the RV we could assume that the simulated anisotropy ratios would be higher than the experimental ones, similar to the results obtained in the LV.

Additionally, ventricular wall movement was not included in the model. Although wall motion can affect the catheter's contact with the epicardium, plastic holders were used experimentally to stabilize the electrode–tissue contact and preserve its near perpendicular orientation, justifying the decision to ignore wall motion in this case. The contact was assumed to be rigid, although elastic deformation occurs in the tissue because of the catheter interaction. However, the impact on the contact area for cylindrical electrodes, such as the one used in this study, is minimal.^21^ Investigating the effects of elastic deformation is also part of our ongoing work.

Finally, this work considers a thoracic geometry of a porcine animal, which might introduce some alterations of the electric field. The quantification of such changes requires the creation of the geometry of the full animal and goes beyond the scope of the existing work. The open-chest computational geometry was designed to visually match the experimental model in Figure 1. Median sternotomy may alter the thorax of the animal, which is not considered in the presented model geometry.

## Conclusions

A single lethal electric field threshold is insufficient to accurately characterize both lesion depth and width in cardiac PFA, using a focal catheter and a neutral electrode. Our study suggests the use of 2 thresholds for the prediction of the IRE area in such PFA setups on ventricular tissues. For the given PFA waveforms, a threshold between 270 and 500 V/cm provides satisfactory results for lesion depth, whereas a higher threshold between 790 and 1000 V/cm better captures the lesion width.
