# Supplementary material for: Is a single lethal electric field threshold sufficient to characterize the lesion size in computational modeling of cardiac pulsed-field ablation?
Source: Heart Rhythm O2. 2025 Feb 22;6(5):671–7. doi: 10.1016/j.hroo.2025.02.014 (PMC12147587; doi:10.1016/j.hroo.2025.02.014)
Supplement: Supplementary Material 1 [file mmc1.pdf]

Supplementary material

Is a single lethal electric field threshold sufficient to characterize the lesion size in computational modeling of cardiac pulsed field ablation?

Argyrios Petras   Gerard Amoros Figueras   Zoraida Moreno Weidmann
Tomás García-Sánchez   David Viladés Medel   Antoni Ivorra
José M. Guerra   Luca Gerardo-Giorda

Geometry generation

The model geometry was segmented manually using Slicer3D software (`www.slicer.org`). The segmentation includes the porcine torso, bones, shoulder blades, costal cartilage, sternum, lungs, main blood vessels (superior and inferior vena cavae, aorta, pulmonary artery, pulmonary veins), four-chamber heart and pericardium. In the case of tissues not visible by the CT scan (particularly for the atrial walls and pericardium), average anatomical thicknesses were considered in the geometry generation [8, 3, 16].

The segmented geometry was meshed via in-house semi-automatic scripts using the Studio software by NumeriCor GmbH (`www.numericor.at`), which is capable of providing high quality meshes following the segmented image. The catheter was designed and meshed using Salome software (`www.salome-platform.org`), and embedded to the geometry using Studio. The catheter was placed on 5 different positions on the epicardial surface of the left and right ventricles (as shown in Figure 1 of the main manuscript), following the normal direction of the desired mesh node, at a depth of 0.4 mm. The near perpendicular placement is also supported by the experimental setup, during which the shown placeholders in Figure 1 kept the catheter at a near perpendicular orientation.

To mimic the open-chest experimental setup, a portion of the frontal torso of the porcine geometry (including costal cartilage, sternum and part of pericardium) was tagged as air (see Figure 1). The retagged part was chosen to visually match the experimental setup, as seen in Figure 1 of the main manuscript.

Mathematical model

The electric field is identified using the equation

$$\nabla \cdot (\sigma(\mathbf{E})\nabla\Phi) = 0,$$

where $\sigma(\cdot)$ is the electrical conductivity that depends on the electric field $\mathbf{E} = \nabla\Phi$ and Φ is the electrical potential. Following [6], a sigmoidal dependence on the electric field intensity is assumed for the electrical conductivity, to model the increase of its value due to irreversible electroporation, given by

$$\sigma(\mathbf{E}) = \sigma_0 + \Delta\sigma \exp\left(-\exp\left(-\alpha(|\mathbf{E}| - E_{thr})\right)\right),$$

where σ_0 is the electrical conductivity prior to electroporation, $\Delta\sigma$ is the difference in the conductivity before and after electroporation, α is a scaling constant and E_{thr} is the electric field lethal threshold.

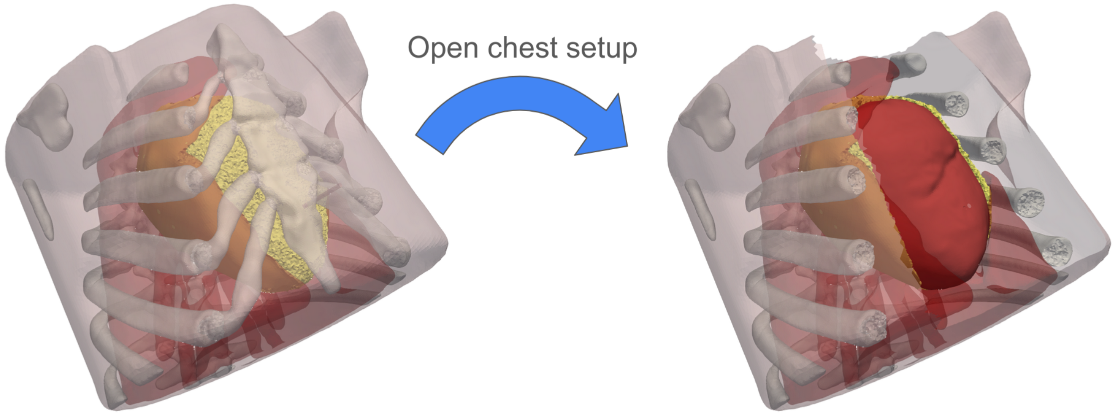

Figure 1: The transition from the segmented geometry of the porcine CT scan to the open-chest geometry that mimics the experimental setup.

Parameters

Extra care needs to be taken in the parametrization of the computational models, in order to closely match experiments and avoid the creation of digital chimera. The experimental model uses a porcine animal, thus porcine conductivity values should be considered for all the different subdomains of the model geometry. For this reason, we do not use the ITIS database [7] to obtain the tissue parameters of our model, since the values in this database refer to human biophysical tissue properties.

To dwell deeper in this aspect, the blood electrical conductivity depends on the level of the hematocrit of the blood [12] and the blood flow pulsatility [17] among other factors. The blood conductivity given in [7] refers to human blood, while there is evidence that different species have different blood properties [9]. Thus, we selected the corresponding average value (average per pulsatility cycle) for a porcine blood, given in [17].

Similarly, the model in [6] simulates a rat heart, and there is also evidence of interspecies differences in the cardiac tissue electrical properties [5]. We consider the value from [15], which is measured for in-vivo porcine hearts. All the other parameters also consider porcine values, as summarized in Table 1.

For the torso, a ratio of 82:18 muscle:fat is considered, following [11], while the lungs use a 50:50 inflated:deflated ratio. The porcine pericardium consists of a fibrous and a serum part, similar to other mammals [16]. The fibrous part is composed largely by collagen type I [18]. The electrical conductivity of different concentrations of collagen solutions has been reported between $0.02 - 0.08\,\mathrm{S/m}$ [2]. On the other hand, the pericardial fluid contains largely Na^+ and Cl^- [16]. Typical experiments use fluids like saline or plasma for pericardial effusions [10], which have a large conductivity of $1.2\,\mathrm{S/m}$ [14]. Since no exact parameter value was found in the literature for the electrical conductivity of the pericardium, and by taking into account that we are modeling an open-chest experimental setup in which some parts are exposed to air, it is reasonable to assume a more resistive value, such as the one reported in Table 1. Nonetheless, by conducting a parameter sensitivity study for the value of the electrical conductivity of the pericardium, by exploring different values from $0.1\,\mathrm{S/m}$ up to $1\,\mathrm{S/m}$, negligible differences were observed in the lesion morphology, as expected due to the open-chest setup.

All values are considered at a frequency of 100 kHz, to model the pulse frequency of 90 kHz used in the experimental setup.

The scaling factor $\alpha = 10^{-4}\,\mathrm{m/V}$ and the electrical conductivity difference value $\Delta\sigma = 0.14\,\mathrm{S/m}$ were chosen following [6] for 90 kHz. Different values for E_{thr} were examined in the range of 200 and 1000 V/cm.

Type	Value (S/m)	Reference
Torso	0.264	[4]
Bones	10^{-2}	[1]
Lungs	0.12	[4]
Blood	1.2	[17]
Cardiac tissue (σ_0)	0.44	[15]
Pericardium	0.1	
Electrode	4.1×10^{7}	[13]
Thermistor	10^{-5}	[13]
Air	10^{-12}	

Table 1: Summary of the electrical conductivity parameters of our computational model

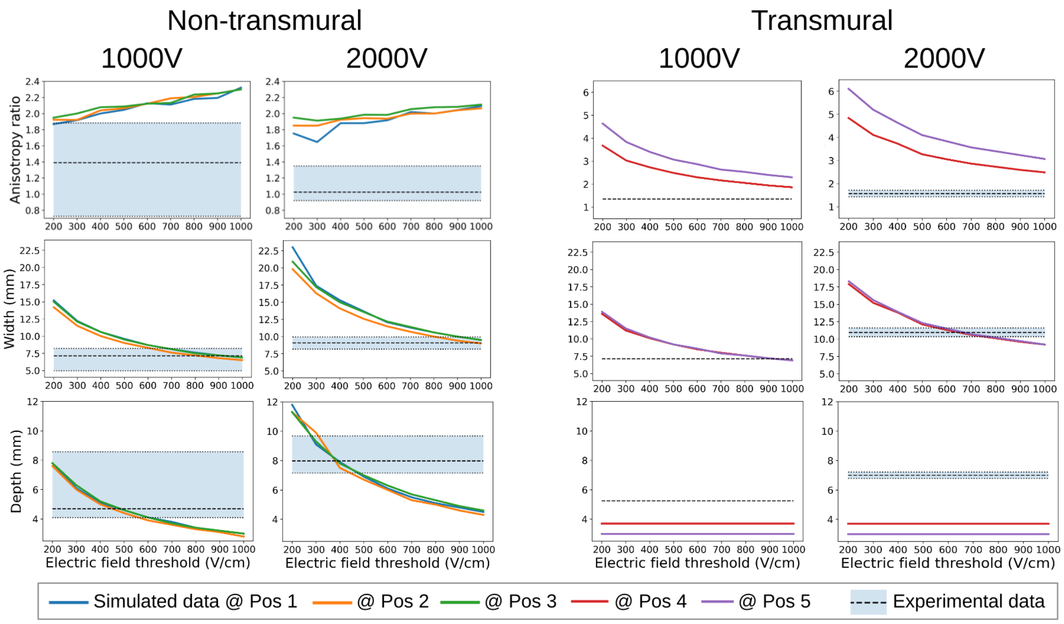

Figure 2: The lesion depth, width and aspect ratio of the experimental and simulated transmural and non-transmural lesions, for different peak voltages. In colored solid lines are the simulated data and in the shaded area and dashed line the experimental data range and the corresponding median value.

Anisotropy ratio for transmural lesions

The anisotropy ratio is a good measure when describing the morphology for non-transmural lesions, however for transmural lesions the wall thickness greatly affects this measure, making it unreliable for such lesions. As shown in Figure 2, the anisotropy ratio for transmural lesions has a completely opposite trend to the non-transmural ones for different lethal thresholds, following the trend of the lesion width. Indeed, despite having a comparable lesion width for simulated lesions at the positions 4 and 5, the anisotropy ratio of the 2 appears quite different due to the different tissue thickness. Thus, unless the tissues have a comparable wall thickness, it is not recommended to use the anisotropy ratio for the identification of the morphology of transmural lesions, but rather the width.

Data availability

The simulation results can be provided to the interested readers upon reasonable request. Further details for the model geometry can be provided by contacting the corresponding author of this

work.

References

[1] Bilal Amin, Muhammad Adnan Elahi, Atif Shahzad, Emily Porter, and Martin O'Halloran. A review of the dielectric properties of the bone for low frequency medical technologies. *Biomedical Physics & Engineering Express*, 5(2):022001, jan 2019.

[2] M Ashoorirad, M Saviz, and A Fallah. On the electrical properties of collagen macromolecule solutions: Role of collagen-water interactions. *Journal of Molecular Liquids*, 300:112344, 2020.

[3] Channing Convelbo, Hanane El Hafci, Hervé Petite, and Rachid Zegdi. Traumatic leaflet injury: comparison of porcine leaflet self-expandable and bovine leaflet balloon-expandable prostheses. *European Journal of Cardio-Thoracic Surgery*, 53(5):1062–1067, 2018.

[4] C Gabriel, A Peyman, and E H Grant. Electrical conductivity of tissue at frequencies below 1 mhz. *Physics in Medicine & Biology*, 54(16):4863, jul 2009.

[5] Camelia Gabriel, Azadeh Peyman, and Edwin H Grant. Electrical conductivity of tissue at frequencies below 1 mhz. *Physics in medicine & biology*, 54(16):4863, 2009.

[6] Tomás García-Sánchez, Gerard Amorós-Figueras, Esther Jorge, María C. Campos, Elad Maor, Jose M. Guerra, and Antoni Ivorra. Parametric study of pulsed field ablation with biphasic waveforms in an in vivo heart model: The role of frequency. *Circulation: Arrhythmia and Electrophysiology*, 15:693–705, 10 2022.

[7] P Hasgall, F Di Gennaro, C Baumgartner, E Neufeld, B Lloyd, MC Gosselin, D Payne, A Klingenböck, and N Kuster. IT'IS Database for thermal and electromagnetic parameters of biological tissues, 2022.

[8] Susumu Ishikawa, Shigeru Oki, Masato Muraoka, Kiyohiro Oshima, Kenji Kashiwabara, and Yasuo Morishita. Epicardial radiofrequency ablation on a beating heart: an experimental study. *Ann Thorac Cardiovasc Surg*, 11(1), 2005.

[9] Frédéric Jaspard and Mustapha Nadi. Dielectric properties of blood: an investigation of temperature dependence. *Physiological measurement*, 23(3):547, 2002.

[10] Canan M Karatay, Thomas Fruehan, George W Lighty, Robert M Spear, and Harold Smulyan. Acute pericardial distension in pigs: effect of fluid conductance on body surface electrocardiogram qrs size, 1993.

[11] A. D. Mitchell, J. M. Conway, and W.J.E. Potts. Body composition analysis of pigs by dual-energy x-ray absorptiometry2. *Journal of Animal Science*, 74(11):2663–2671, 11 1996.

[12] Amalric Montalibet, J Jossinet, A Matias, and D Cathignol. Electric current generated by ultrasonically induced lorentz force in biological media. *Medical and Biological Engineering and Computing*, 39:15–20, 2001.

[13] Argyrios Petras, Massimiliano Leoni, Jose M. Guerra, Johan Jansson, and Luca Gerardo-Giorda. A computational model of open-irrigated radiofrequency catheter ablation accounting for mechanical properties of the cardiac tissue. *International Journal for Numerical Methods in Biomedical Engineering*, pages 1–20, 10 2019.

[14] Richard Sauerheber and Bettina Heinz. Temperature effects on conductivity of seawater and physiologic saline, mechanism and significance. *Chem. Sci. J*, 6(109):4172, 2015.

[15] Jang-Zern Tsai, J.A. Will, S. Hubbard-Van Stelle, Hong Cao, S. Tungjitkusolmun, Young Bin Choy, D. Haemmerich, V.R. Vorperian, and J.G. Webster. In-vivo measurement of swine myocardial resistivity. *IEEE Transactions on Biomedical Engineering*, 49(5):472–483, 2002.

[16] Konstantinos Vogiatzidis, Sotirios G. Zarogiannis, Isaac Aidonidis, Evgeniy I. Solenov, Paschalis Adam Molyvdas, Konstantinos I. Gourgoulianis, and Chrissi Hatzoglou. Physiology of pericardial fluid production and drainage, 2015.

[17] Jianming Wen, Nen Wan, Huilu Bao, and Jianping Li. Quantitative measurement and evaluation of red blood cell aggregation in normal blood based on a modified hanai equation. *Sensors*, 19(5), 2019.

[18] Sabra Zouhair, Eleonora Dal Sasso, Sugat R Tuladhar, Catia Fidalgo, Luca Vedovelli, Andrea Filippi, Giulia Borile, Andrea Bagno, Massimo Marchesan, Giorgio De Rossi, et al. A comprehensive comparison of bovine and porcine decellularized pericardia: new insights for surgical applications. *Biomolecules*, 10(3):371, 2020.
