# Supplementary material for: Is a single lethal electric field threshold sufficient to characterize the lesion size in computational modeling of cardiac pulsed-field ablation?
Source: Heart Rhythm O2. 2025 Feb 22;6(5):671–7. doi: 10.1016/j.hroo.2025.02.014 (PMC12147587; doi:10.1016/j.hroo.2025.02.014)
Supplement: Supplementary Material 2 [file mmc2.pdf]

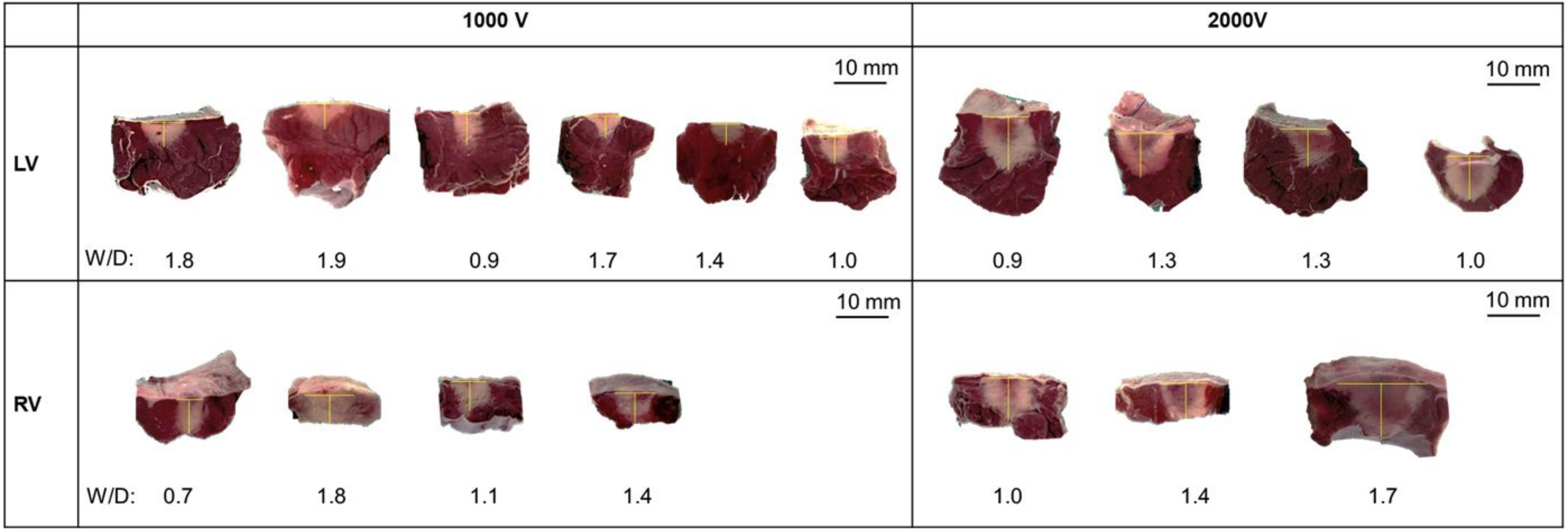

Supplementary Figure 1. Macro photographs of the experimental PFA ablation lesions for the different applied voltage levels (1000 and 2000V) in the LV and RV. Each lesion has the width and depth delimited and its anisotropy ratio (W/D) below.
